# Effect of riboflavin and roseoflavin, a structural analogue of riboflavin, on the growth of selected microbial isolates from human saliva using a riboflavin-free cultivation medium

**DOI:** 10.1007/s12223-025-01317-w

**Published:** 2025-09-17

**Authors:** Nadja Schwendenmann, Sophie Burger, Leonie Steiner, Lars Seufert, Matthias Kohl, Matthias Mack, Markus Egert

**Affiliations:** 1 Faculty of Health, Medical and Life Sciences, Microbiology and Hygiene Group, Institute of Precision Medicine, Furtwangen University of Applied Sciences, Villingen-Schwenningen, Germany; 2 Institute of Technical Microbiology, Mannheim University of Applied Sciences, Mannheim, Germany; 3 Faculty of Health, Medical and Life Sciences, Bioinformatics and Biostatistics Group, Institute of Precision Medicine, Furtwangen University of Applied Sciences, Villingen-Schwenningen, Germany

**Keywords:** Oral microbiota, *Streptococcus*, Riboflavin, Roseoflavin, Antivitamin

## Abstract

The antivitamin roseoflavin (RoF) is a structural analogue and competitive inhibitor of riboflavin (RF, vitamin B_2_). Stimulated by a recent report that cariogenic *Streptococcus mutans* is prototrophic for RF, we conducted pilot investigations on the effect of RF and RoF on the growth of selected microbial isolates from human saliva to lay a first foundation whether oral application of RoF might be suitable to modify the human oral microbiota for the sake of human health. Microbial isolates from saliva samples of 41 healthy volunteers were obtained and identified by matrix-assisted laser desorption/ionization time-of-flight mass spectrometry. Thirty different microbial isolates were investigated regarding growth promotion by RF and susceptibility to RoF using growth experiments. Isolates were grown in RF-free medium without supplements or supplemented with 100 µmol/L RF, 100 µmol/L RoF, or a combination of both, respectively. Absorbance (A) was measured before and after 24 h of incubation using a spectrophotometer. Log2-transformed absorbance data were analyzed using analysis of covariance (ANCOVA). Mainly the growth of gram-positive bacteria was affected by RF and RoF. Growth of 5 gram-positive species from the genera *Enterococcus*, *Staphylococcus*, and *Streptococcus* was significantly reduced upon addition of RoF when compared to growth without any additives. Simultaneous administration of RF and RoF led to significantly reduced growth of at least 3 gram-positive species from the genera *Bacillus* and *Staphylococcus* when compared to growth with RF alone. Growth of cariogenic *S. mutans* was significantly enhanced by RF, and this effect was at least partly, but not significantly, reversed by supplementing the growth media with RoF. In agreement with previous investigations, our data suggests that gram-positive bacteria in the human oral cavity might be more affected by RoF than gram-negatives. Our future research aims at characterizing the inhibited bacteria, the underlying molecular mechanism(s), and the effect of RoF on the composition of the complex microbial community in the human oral cavity in more detail.

**Supplementary Information:**

The online version contains supplementary material available at https://doi.org/10.1007/s12223-025-01317-w.

## Introduction

The human oral microbiota is comprised of more than 700 species of microorganisms, some of which have been shown to cause a number of oral and systemic diseases, such as caries, periodontitis, and cardiovascular disease (Dewhirst et al. 2010; Sanz et al. 2020). In healthy humans, a homeostatic balance between the host and the oral microbiota is maintained by various regulatory mechanisms. Disease mainly occurs when this homeostatic balance of the oral microbiota is disrupted (Lamont and Hajishengallis 2015). If dysbiosis occurs and disease-causing microbial species increase in abundance, anti-infectives can be used to diminish certain microbial subgroups. For bacterial pathogens, many different anti-infectives (e.g., antibiotics) are available. However, due to the steady increase of multidrug-resistant strains, new approaches are needed as a routine application of antibiotics should not take place (Hutchings et al. 2019). Promising and still underexplored candidates may be antivitamins: compounds that prevent the essential effects of vitamins (Somogyi and Trautner 1974). Class A antivitamins are inhibitors of vitamin biosynthesis, vitamin transportation, and vitamin-dependent enzymes, while class B antivitamins are defined as structural modifiers (Zelder et al. 2015). So far, mostly antivitamins of vitamin B_9_ and vitamin K have been approved and used in a human clinical context, most notably perhaps the antivitamin K warfarin and the antivitamin B_9_ methotrexate (Knox et al. 2024). For microbiome studies, other vitamin antagonists might be a promising approach to modulate the composition of the microbiota and serve as novel antimicrobial agents.

**Fig. 1:**
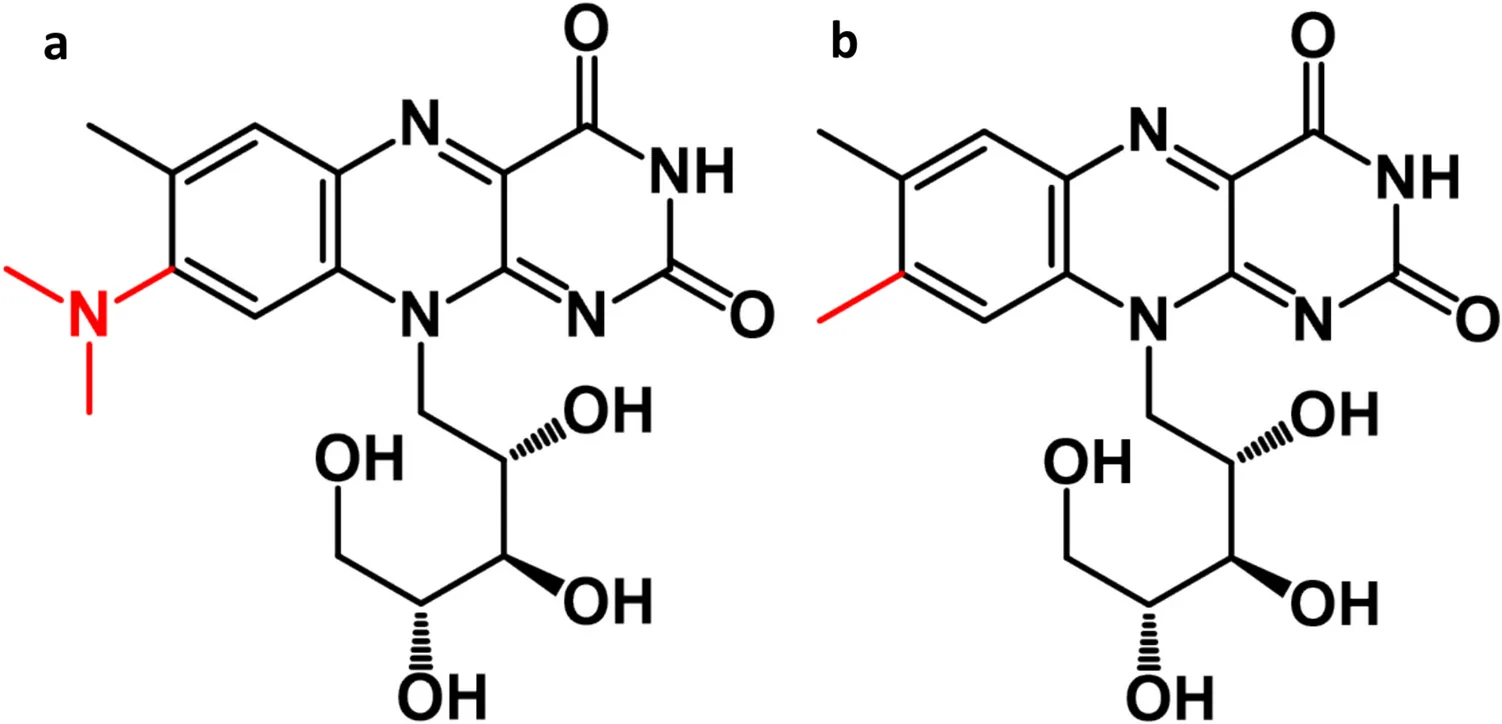
Chemical structure of roseoflavin (**a**) and riboflavin (**b**). The dimethylamino group in RoF and the methyl group in RF are highlighted in red, respectively. Image source: M. Mack

In soil, *Streptomyces davaonensis*, *Streptomyces cinnabarinus*, and *Streptomyces berlinensis* produce a bright red pigment called roseoflavin (RoF; 8-demethyl-8-dimethylamino-riboflavin) (Liunardo et al. 2024; Otani et al. 1974; Schwarz et al. 2016). RoF is a structural riboflavin (RF, vitamin B_2_) analogue and competitive inhibitor of RF and is metabolized by the same enzymes as RF (Vogl et al. 2007). In contrast to RF, RoF does not have a methyl group at position 8, but a dimethylamino group (Fig. 1).

Instead of the functional flavin cofactors flavin mononucleotide (FMN) and flavin adenine nucleotide (FAD), RoF is converted to roseoflavin mononucleotide (RoFMN) by riboflavin flavokinase (EC 2.7.1.26) and roseoflavin adenine dinucleotide (RoFAD) by FAD synthetase (EC 2.7.7.2) (Grill et al. 2008). RoFMN and RoFAD have the potential to negatively affect flavoproteins, thus exerting antibiotic activity (Matern et al. 2016). In addition, RoFMN targets FMN riboswitches, i.e., genetic elements which regulate RF biosynthesis and transport (Pedrolli and Mack 2014). Interestingly, the dimethylamino group of RoF seems to permit better binding abilities than the methyl group of RF to the riboswitch aptamer structure (Lee et al. 2009; Pedrolli et al. 2014). Other flavin analogues, such as 8-demethyl-8-aminoriboflavin (AF), 5FDQD, and ribocil-C possess antimicrobial properties as well (Blount et al. 2015; Howe et al. 2015; Kasai et al. 1978; Pedrolli et al. 2013). The mechanism of action is largely the same as for RoF (Pedrolli et al. 2011).

The endogenous synthesis of riboflavin is referred to as the riboflavin biosynthetic pathway (RBP) and has been extensively studied in several species, including *Bacillus subtilis* (Lee et al. 2009; Liu et al. 2023). Many known gram-negative bacteria rely on endogenic RF synthesis and are unable to take up exogenic RF, as they lack the necessary transporters. On the other hand, gram-positive bacteria appear to be able to both synthesize their own RF and also take up RF from the environment. Gram-positive lactic acid bacteria, in turn, are entirely reliant on exogenous RF and unable to synthesize RF (Gutiérrez-Preciado et al. 2015).

Because RoF is taken up via RF transporters, gram-positive bacteria in general are susceptible to RoF (Gutiérrez-Preciado et al. 2015). A recent study revealed that the gram-positive caries pathogen *S. mutans* possesses SMU.1703c, a riboflavin transporter, while not having any RF biosynthetic enzymes (Gutiérrez-Preciado et al. 2015; Jijakli and Jensen 2019; Turner et al. 2020). Therefore, *S. mutans* might be susceptible to RoF. This might open up new therapeutic approaches using RF analogues, such as RoF, in the future.

So far, an antimicrobial effect of RoF has been demonstrated against *Plasmodium falciparum* in vitro and in vivo (Hemasa et al. 2022). Additionally, it has been shown that RoF inhibits growth of *Listeria monocytogenes*, but also enhances virulence (Mansjö and Johansson 2011; Matern et al. 2016).

Although the anti-infective potential of RoF was already recognized, this substance has, to the best of our knowledge, not yet been investigated for its influence on complex microbial communities of human origin (Pedrolli et al. 2014, 2013). Searching the pubmed database on 04–03-2025 for “roseoflavin AND microbiota” yielded a single study, in which RoF was used to select for RF-overproducing lactic acid bacteria (Mohedano et al. 2019).

Here, we conducted growth experiments with a selection of isolated oral microbes, mostly stemming from saliva samples of healthy volunteers, in RF-free medium supplemented with RF and/or RoF. Our study aims at providing initial data to evaluate whether RoF treatment has the general potential to modify the human oral microbiota for the sake of human health, e.g., by inhibiting the growth of *S. mutans*.

## Material and methods

### Sample collection and processing

Saliva samples were obtained from 41 healthy human volunteers (co-workers and students of Furtwangen University; 29 females, 12 males), who reported having no carious lesions, periodontitis, or similar oral diseases at the time of sample collection, based on the most recent information provided to them by their dentist. The mean age of the probands was 27.8 years. Volunteers had not taken any antibiotics 3 months prior to sample collection and were asked not to eat or drink at least 1 h prior to sample collection. Saliva was collected by spitting into sterile, nuclease-free 1.5 mL reaction tubes and processed immediately. For saliva collection, volunteers were asked to rub their tongue on all mouth surfaces and accumulate saliva for 1 min. Afterwards, they were asked to collect between 700 and 800 µL saliva into the reaction tubes. To obtain pure cultures on agar plates, the saliva was diluted 1:100 and 1:1000 with sterile 0.9% sodium chloride solution.

### Cultivation and isolation of different microbial species

Immediately after sample dilution, 10 µL of diluted saliva samples were streaked onto agar plates and incubated at 37 °C for 24 to 48 h. For aerobic incubation, LB-Lennox agar (Carl Roth, Karlsruhe, Germany) and tryptic soy agar (VWR, Darmstadt, Germany) were used. Columbia blood agar supplemented with 5% defibrinated sheep blood (Fisher Scientific, Schwerte, Germany) and chocolate agar supplemented with Vitox (Fisher Scientific) were used for anaerobic incubation in an anaerobic jar. After incubation, single colonies were isolated with a sterile toothpick, transferred into fresh brain heart infusion (BHI) broth, and incubated at 37 °C for another 24 h, either aerobically or anaerobically. Afterwards, the isolated pure cultures were incubated on solid medium again for subsequent identification.

### Identification of microbial pure cultures with MALDI-TOF MS

A single colony from the microbial pure culture was transferred onto a stainless steel MALDI target plate (Bruker Daltonics, Bremen, Germany). Each colony was applied to two separate spots on the target plate for duplicate analysis. After a drying period of 1 min, each spot was layered with 1 µL α-cyano-4-hydroxycinnamic acid (HCCA; Bruker Daltonics) and left to dry for another 2 min. Isolated pure cultures were identified via MALDI-TOF MS (MALDI Biotyper IVD System, Bruker Daltonics), using the following software packages: flexControl 3.4 (Version 204.10), MBT Compass (Version 4.1.100), and MALDI Biotyper Compass database bacteria and filamentous fungi (Version 4.1.14) (all Bruker Daltonics).

For the measurement and identification of microorganisms, positive linear mode (ion source 1, 20.04 kV; ion source 2, 18.33 kV; lens, 6.02 kV; laser frequency, 60 Hz) was used. For each spectrum, 240 laser shots in 40-shot steps were measured and acquired using the MBT AutoX settings. According to manufacturer’s specifications, identification scores of ≥ 2.0 were considered reliable for species identification (Cuénod et al. 2021; Sauer et al. 2008). If the score after MALDI-TOF MS identification was < 2.0, single colonies were transferred into BHI broth again and fresh pure cultures were incubated on agar plates to repeat the analysis. Samples with identification scores still lower than 2.0 were considered unreliable for species identification and thus not taken into further consideration. Finally, identification on species level was only considered to be secure when the list of species provided by the Bruker software was clearly headed by one species. Afterwards, cultures with a score ≥ 2.0 were used for preparation of 25% glycerol-containing cryogenic cultures and stored at − 80 °C.

### Effects of riboflavin and/or roseoflavin addition on microbial growth

To test the isolated and identified microorganisms for growth stimulation by RF and inhibition by RoF, an agar-free and riboflavin-free medium (Riboflavin Assay Medium, RFAM; Becton Dickinson, Heidelberg, Germany) was used.

First, bacteria from the strain collection were grown to a high density in BHI at 37 °C, either aerobically or anaerobically. Then, 2 mL of each liquid culture was transferred to a reaction tube and centrifuged for 5 min at 10,000 g. The supernatant was discarded and the pellet resuspended in 1 mL 1 × phosphate-buffered saline (PBS) and centrifuged again. This step was repeated three times to ensure that no RF from the BHI was carried over. After the third wash step, the pellet was resuspended in 1 mL RFAM.

The bacterial suspensions were then diluted with RFAM to visually match the turbidity of 0.25 McFarland barium sulfate suspension. We employed the McFarland 0.25 standard to approximate uniformity in microbial density prior to initiating the experiment. This standard was selected to ensure that microbial populations would not enter the stationary phase within the 24 h of growth. Post-incubation spectrophotometric readings recorded at 0 and 24 h were normalized to account for and mitigate any minor variations in microbial load.

For each treatment, 2 mL of the diluted bacterial suspension were transferred to a fresh reaction tube, where RF, RoF, or both in combination were added to final concentrations of 100 µmol/L, respectively. These concentrations were chosen because previous research by Matern et al. (2016) showed sufficient inhibition of *L. monocytogenes* using 100 µmol/L RoF, while treatment with 100 µmol/L RF did not negatively affect bacterial growth. Immediately after that, the first absorbance measurement was carried out using a spectrophotometer (SPEKOL 1500, Analytik Jena, Jena, Germany) at 600 nm (A_600_). The second half of the bacterial suspension was incubated for 24 h at 37 °C aerobically or anaerobically. Finally, A_600_ of the incubated bacterial suspensions was measured again. For each isolate, the different incubation conditions were tested three times independently (*n* = 3, each). Raw data are available in Online Resource 1.

### Data analysis

Absorbance data stemming from RF stimulation and RoF sensitivity testing were analyzed with R (Version 4.3.2) and RStudio. To find out whether growth in RFAM within 24 h was significant, the log2 fold change (log2FC) was calculated for each microbial species using the A_600_ data at 0 and 24 h in RFAM without any supplementation. We performed quantile–quantile plots to check normality and found no clear deviations (Lüdecke et al. 2021). Pairwise *t*-tests were employed to determine whether the log2-transformed A_600_ values before and after growth differed significantly. Growth differences among the four cultivation conditions were analyzed for each microbial species using analysis of covariance (ANCOVA) and Tukey’s HSD test for the log2 A_600_ measurements at 24 h, which were adjusted to the corresponding log2 A_600_ measurements at 0 h (Senn 2006).

## Results

MALDI-biotyping of 321 isolated microorganisms from 41 healthy volunteers revealed 37 different bacterial and one fungal species. 56.0% of the isolated microorganisms were gram-positive, and 43.0% were gram-negative. The most commonly identified genera were *Streptococcus* (41.4%), *Neisseria* (23.1%), and *Staphylococcus* (8.4%).

Out of the 38 identified microbial species, 27 bacterial and one fungal species were successfully cultivated and added to a proprietary strain collection (Online Resource 2). In addition, *S. mutans* (DSM 20523) and *B. subtilis* (DSM 10) were obtained from the German Collection of Microorganisms and Cell Cultures. *S. mutans* was added to our strain collection because it is an important caries pathogen, but we were unable to isolate it from the saliva samples. This might be due to the selection of a relatively small number of healthy volunteers explicitly confirmed to be free of any oral diseases, as required by our Ethics Committee. *B. subtilis* was added to the strain collection as another control organism.

**Fig. 2:**
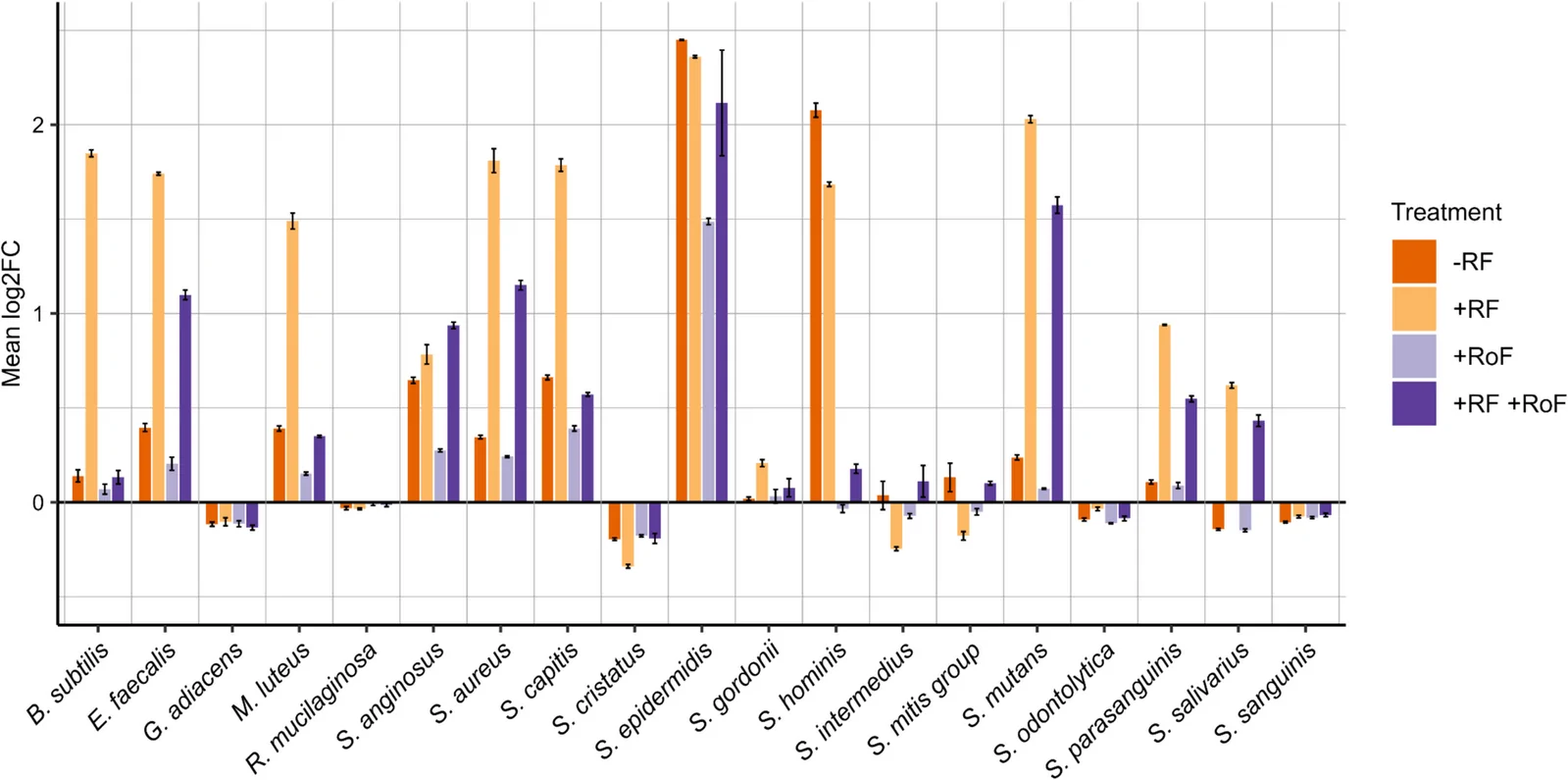
Effects of riboflavin (RF) and roseoflavin (RoF) on gram-positive bacteria isolated from human saliva. The microorganisms were grown in BHI medium, transferred into riboflavin assay medium, and incubated without any additions, with 100 µmol/L RF, with 100 µmol/L RoF, and with 100 µmol/L of both RF and RoF for 24 h at 37 °C, respectively. Values are shown as mean log2 fold changes ± SEM (*n* = 3)

**Fig. 3:**
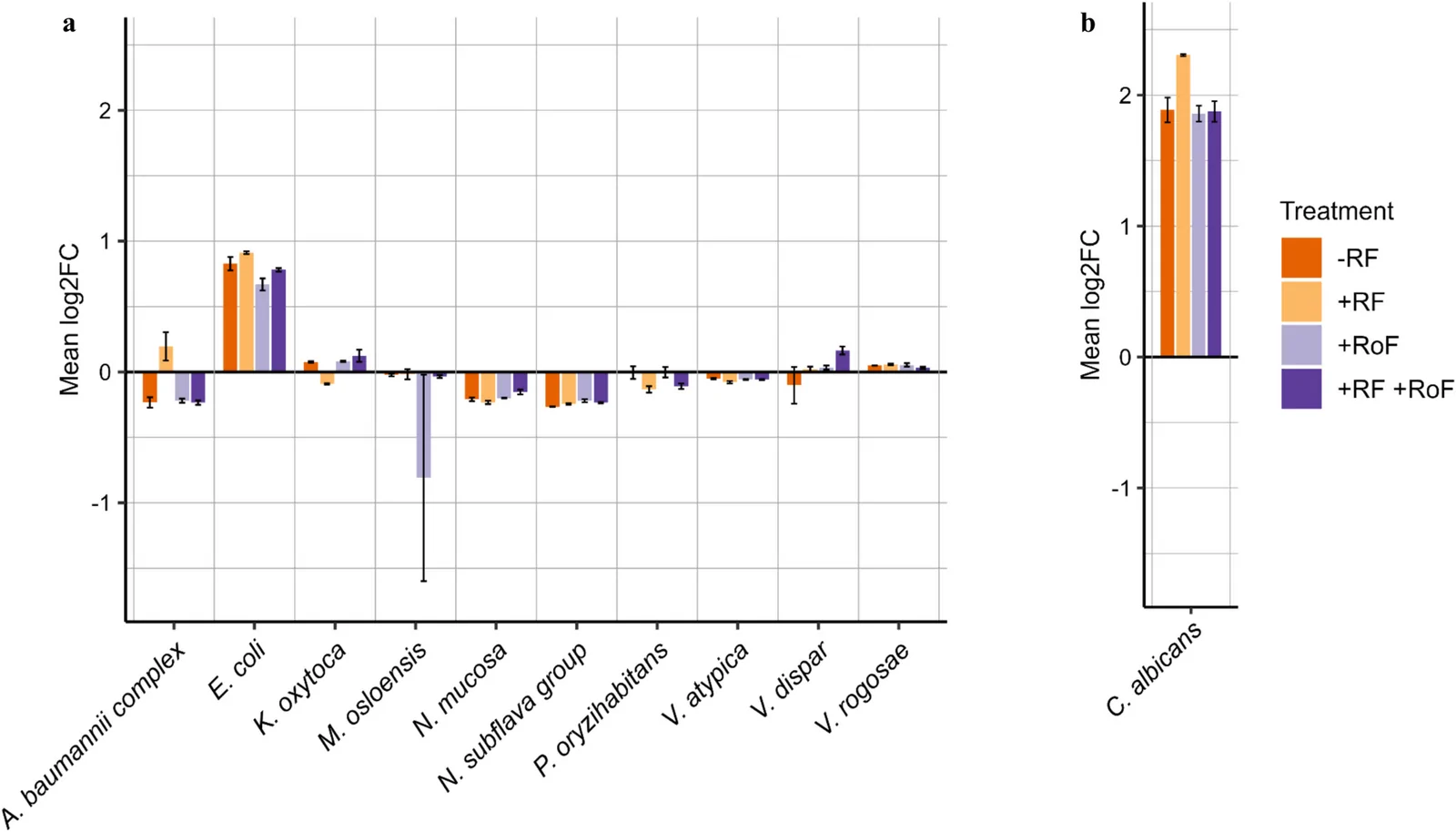
Effects of riboflavin (RF) and roseoflavin (RoF) on gram-negative bacteria (**a**) and fungi (**b**) isolated from human saliva. For further details, see legend of Fig. 2

The final strain collection was comprised of 29 bacterial and one fungal species. The securely identified species were then tested for the effects of added RF and/or RoF on their growth using absorbance measurements. Figure 2 shows the results for gram-positive bacteria, while Fig. 3 displays the results for gram-negative bacteria and the yeast *Candida albicans*.

Of the 19 tested gram-positive species, 10 showed significant growth in RFAM without any additives (orange bars) over the span of 24 h.

Additionally, seven gram-positive species grew significantly better with added RF (yellow bars) compared to growth in RFAM without any additives, i.e., *B. subtilis*, *Staphylococcus aureus*, *Streptococcus gordonii*, *S. mutans*, *Streptococcus parasanguinis*, *Streptococcus salivarius*, and *Streptococcus sanguinis*. Interestingly, *Schaalia odontolytica*, *Staphylococcus hominis*, *Streptococcus intermedius*, and *Streptococcus mitis* group grew significantly worse with added RF.

Growth of 5 gram-positives (*Enterococcus faecalis*, *S. aureus*, *Staphylococcus capitis*, *S. hominis*, *S. mitis* group) was significantly reduced upon the addition of RoF (light purple bars) when compared to growth without any additives. Simultaneous administration of RF and RoF (dark purple bars) led to significantly reduced growth in the cases of *B. subtilis*, *S. aureus*, and *S. hominis*, while *S. mitis* group and *S. sanguinis* grew even slightly, but significantly better in comparison to growth with added RF.

*E. faecalis*, *Micrococcus luteus*, *S. capitis*, *Staphylococcus epidermidis*, *S. mutans*, *S. odontolytica*, *S. parasanguinis*, and *S. salivarius* also showed reduced growth when RF and RoF were simultaneously added; however, the difference to growth with RF alone was not statistically significant under the test conditions applied here.

Of the 10 tested gram-negative species, 2 showed significant growth in RFAM without any additives (orange bars) over the span of 24 h. Only *Veillonella dispar* grew significantly better with added RF (yellow bars), while only *Pseudomonas oryzihabitans* grew significantly worse. None of the gram-negatives showed significantly reduced growth upon RoF addition (light purple bars). With the exception of *Escherichia coli* and *Klebsiella oxytoca*, the gram-negative isolates did not grow in the RF-free medium used here. However, the addition of RF also did not improve growth. So, the addition of RoF alone or in combination (dark purple bars) was without any inhibitory effect, also in the case of *E. coli* and *K. oxytoca*. Partly, the addition of RoF (alone or in combination) even seemed to improve the bad growth (Fig. 3).

*C. albicans* grew well without RF (orange bars) and even better with added RF (yellow bars). Similar to many gram-positive bacteria, the addition of RoF alone (light purple bars) and in combination with RF (dark purple bars) prevented better growth. However, all these differences were not statistically significant.

To facilitate comparisons, significance levels for all statistically significant comparisons are summarized in Table 1.

**Table 1:** Significance levels of different growth conditions for selected bacteria. ^†^Compared to growth in RFAM. ^‡^Compared to growth in RFAM with added RF. Statistical significance was calculated for adjusted log2 A_600_ measurements at 24 h using ANCOVA (*n* = 3). Significant effects in the expected direction are marked with asterisks corresponding to the *p*-values. Significance levels: ns = *p* ≥ 0.05; * = *p* < 0.05; ** = *p* < 0.01; *** = *p* < 0.001. If a significant effect occurred against expectations, this is additionally noted in parentheses after the asterisk: either “prom.” (promotion) or “inhib.” (inhibition), depending on the direction of the effect

Species	Better growth with added RF^†^	Inhibited growth with added RoF	
		+ RoF^†^	+ RF, + RoF^‡^
*B. subtilis*	***	ns	***
*E. faecalis*	ns	*	ns
*N. subflava* group	ns	* (prom.)	ns
*P. oryzihabitans*	* (inhib.)	ns	ns
*S. odontolytica*	** (inhib.)	ns	ns
*S. aureus*	*	**	**
*S. capitis*	ns	**	ns
*S. hominis*	* (inhib.)	***	***
*S. gordonii*	**	ns	ns
*S. intermedius*	* (inhib.)	ns	ns
*S. mutans*	*	ns	ns
*S. mitis* group	*** (inhib.)	*	*** (prom.)
*S. parasanguinis*	***	ns	ns
*S. salivarius*	***	ns	ns
*S. sanguinis*	***	*** (prom.)	** (prom.)
*V. atypica*	ns	ns	* (prom.)
*V. dispar*	***	** (prom.)	ns

First, the growth with added RF was compared to growth in RFAM without any additions to investigate a potential stimulation by RF. The results range from significant growth inhibition, e.g., in *S. mitis* group, to significant growth promotion by RF addition, e.g., in *B. subtilis*. Next, the addition of RoF was statistically examined in comparison to growth in RFAM without any additions. This comparison illustrates the impact of RoF on bacteria that already demonstrated robust growth in RFAM even without any additives, e.g., *S. hominis.* Last, comparisons between growth in RFAM with added RF and growth in RFAM with both RF and RoF were made. This comparison is included because it most closely simulates real-life conditions in the human oral cavity by showing the role of competitive antagonism, although it is a simplified model and does not capture the full complexity of the actual environment.

## Discussion

The aim of our study was to initially investigate the effects of RF and RoF, an RF analogue, on the growth of isolated human oral microorganisms, as a first basis to evaluate the potential of using RoF as a modulator for the human oral microbiota.

Most of the microorganisms isolated in this study belong to the normal human oral microbiota (Dewhirst et al. 2010). Some species are typical environmental bacteria, such as *P. oryzihabitans*, and some species are rather known as members of the human skin microbiome, such as *S. capitis* (Kloos and Schleifer 1975; Kodama et al. 1985). We were unable to isolate *S. mutans* from saliva samples, which is unsurprising considering that previous 16S rRNA gene sequencing studies had revealed a relative abundance of only 0.024% in the saliva of healthy probands (Eren et al. 2014; Escapa et al. 2018; Segata et al. 2012).

In this study and according to the manufacturer’s instructions, a MALDI Biotyper score of ≥ 2.0 is considered sufficient for reliable species identification. Nevertheless, even though many studies have used a score of ≥ 2.0 for secure species identification (Antonation et al. 2020; Baumbach et al. 2021; Cuénod et al. 2021; Harris et al. 2010; Rudrik et al. 2017; Sauer et al. 2008; Sibińska et al. 2024; Sogawa et al. 2024), a score of ≥ 2.3 has been proposed for “highly probable species identification” (Jesumirhewe et al. 2016).

Therefore, it remains possible that some of the species isolated here may have been misidentified. Thus, repeating the experiments using well-defined type strains of the identified microorganisms might be suitable to corroborate our findings.

As RF is contained in probably all conventional cultivation media, including agar, we chose an RF-free medium to test for the effects of added RF and RoF on the microorganisms isolated here. Using media containing RF would most likely yield different results, as the competitive antagonism of RoF and RF would presumably attenuate the antimicrobial effects of RoF, depending, however, on the used concentrations. The used RFAM is defined as completely free of RF, but containing all other nutrients and vitamins that are necessary for the growth of *Lactobacillus rhamnosus*, a gram-positive lactic acid bacterium (Zimbro et al. 2009). Therefore, it is possible that microorganisms tested here might need additional nutrients and vitamins for optimal growth. Thus, the use of RFAM most likely led to some bias, e.g., bad or no growth of some species, and demands follow-up studies including additional media. Socransky et al. (1985) proposed a chemically defined medium for oral microorganisms, which could be adapted to different requirements with a range of RF concentrations in future experiments.

Unfortunately, several isolates did not grow well in the RFAM medium over the course of 24 h; however, some obviously did due to the lack of RF, including *S. mutans*. Better growth with added RF suggests dependency on external RF sources or at least energy saving by using externally supplied instead of self-synthesized RF.

In general, our data suggest that RF and RoF rather affect the gram-positive than the gram-negative members of the oral microbiota, including (opportunistic) human pathogens, such as *S. aureus* and *S. mutans*, though not always significantly (Cheung et al. 2021; Nomura et al. 2020). Previously, *S. aureus* has been reported to have both RF transporters and the riboflavin biosynthetic pathway (RBP) (Gutiérrez-Preciado et al. 2015). In contrast, *S. mutans* and *E. faecalis* have been shown to lack the RBP, but to possess RF transporters (Turner et al. 2020; Gutiérrez-Preciado et al. 2015). In *S. mutans*, deletion of a gene encoding for the RibU-like RF transporter SMU.1703c has been reported to lead to diminished growth, indicating RF auxotrophy (Turner et al. 2020). Such findings suggest that these bacterial species might be susceptible towards the antivitamin RoF. Indeed, all three species behaved very similarly in our study, showing better growth with added RF and less promotion when RF and RoF were supplied in combination. This suggests competitive inhibition of growth by RoF, though not complete inhibition. Since RF is found in a multitude of foods, this may best reflect the actual situation in the oral cavity (Office of Dietary Supplements 2022).

In contrast to gram-negative bacteria, gram-positives generally have riboflavin transporters in addition to the riboflavin biosynthetic pathway to take up RF from the environment (Gutiérrez-Preciado et al. 2015). This can be considered an advantage, as the bacteria are able to take up RF more rapidly and do not spend metabolic energy to synthesize RF de novo. However, RF transporters are also capable of carrying RoF (Vogl et al. 2007). When RoF instead of RF is transported into the cell, the inactive cofactors RoFMN and RoFAD are formed, which leads to the formation of inactive flavoenzymes (Langer et al. 2013). Additionally, RoF was found to bind to FMN riboswitches in *B. subtilis* and thus regulates the expression of associated genes, such as RF transport and synthesis (Lee et al. 2009; Ott et al. 2009). Together, these two mechanisms cause the antibiotic effects of RoF on bacteria, which possess RF transporters.

*S. epidermidis* and *S. hominis* grew very well in the RF-free medium, and in line with this, the addition of even more RF did not promote, but rather slightly inhibited growth. This is in line with previous findings that RF might inhibit or inactivate the growth of different microorganisms through the production of reactive oxygen species (ROS) (Farah et al. 2022; Imlay 2013; Ludwig et al. 2018). We therefore hypothesize that the added RF in combination with potentially self-synthesized RF had a slight inhibitory effect.

RoF significantly inhibited growth, at least for *S. hominis*. As most gram-positive species possess both RF transporters and the RBP, it is possible that both *Staphylococcus* species synthesize sufficient amounts of RF to not show growth stimulation by addition of RF, but also take up certain amounts from the environment, which finally leads to an inhibition by RoF (Gutiérrez-Preciado et al. 2015).

*B. subtilis*, *M. luteus*, *S. capitis*, and *S. parasanguinis* did not grow well in the RF-free medium. However, growth (partly significantly) improved upon addition of RF and was reduced by simultaneously adding RF and RoF. As a consequence, RoF might be an effective antimicrobial agent against these species, too. Some gram-positive species, e.g., *S. epidermidis* and* S. salivarius*, were not significantly inhibited by RoF. Reasons for that may be bad growth in RFAM, unknown resistance mechanisms, or simply the relatively small sample size of our used assay.

Unfortunately, except *E. coli* and *K. oxytoca*, the tested gram-negative species were unable to grow in the RF-free test medium, regardless of added RF, suggesting that other essential growth factors were also absent. The slight, but significant, stimulation of *V. dispar* by RF needs further verification with higher sample sizes, because so far there is no evidence that *V. dispar* possesses RF transporters (Gutiérrez-Preciado et al. 2015). The same holds true for the very slight, but statistically significant growth improvement of RoF or RF/RoF in the case of other *Veillonella* species. Our results are at least in line with the well-known absence of RF transporters in most gram-negative bacteria, so that added RF cannot be utilized (Gutiérrez-Preciado et al. 2015).

Interestingly, the yeast *C. albicans* grew well in the RF-free medium and was promoted by added RF. However, RoF did not inhibit growth but at least reverted the additional growth promotion by RF, suggesting at least some assimilation of RoF.

Of course, it must also be considered that RoF (like most antimicrobial substances) may not only inhibit the growth of potentially pathogenic bacteria but also non-pathogenic microbes or human cells. It has already been shown that RoF is taken up by human cells, where it is converted to RoFMN and RoFAD by human flavokinase and human FAD synthetase, respectively (Pedrolli et al. 2011). Due to the different properties of RoFMN and RoFAD compared to FMN and FAD, this might have negative effects on human metabolism. Therefore, potentially adverse effects of RoF on human cells need to be investigated in future experiments.

In summary, our data suggest that RoF application has the potential to impair the growth of several gram-positive species, including potential pathogens, in the human oral cavity. To substantiate our findings, we plan to conduct additional experiments with other cultivation media and cultivation conditions (e.g., testing a range of RoF concentrations) more similar to the human mouth. Especially a more in-depth investigation of gram-positives that were not susceptible to RoF may be of interest, e.g., by checking for levels of endogenous RF production. Furthermore, we plan cultivation-independent studies to investigate the effects of RF and RoF on the human oral microbiota more broadly, thereby also accounting for yet-uncultivated microbes. We plan to use differential 16S rRNA gene (rDNA) and 16S rRNA sequencing to unravel effects of RF and RoF on the “present” (rDNA-based community composition) and “active” (rRNA-based community composition) fractions of the microbiota in in vitro incubations. Finally, possible impacts of RF and RoF on growth and metabolism of human oral mucosa cells will also be examined to exclude negative effects of RoF on the human host.

## Supplementary Information

Below is the link to the electronic supplementary material.

ESM 1 (XLSX 16 KB)

ESM 2 (DOCX 52 KB)

## Data Availability

Raw data are available in the supplementary material.
