# Supplementary material for: Effect of riboflavin and roseoflavin, a structural analogue of riboflavin, on the growth of selected microbial isolates from human saliva using a riboflavin-free cultivation medium
Source: Folia Microbiol (Praha). 2025 Sep 17;71(5):1359–68. doi: 10.1007/s12223-025-01317-w (PMC13736302; doi:10.1007/s12223-025-01317-w)
Supplement: Supplementary file 1 [file 12223_2025_1317_MOESM2_ESM.docx]

# **Folia Microbiologica**

# **Effect of riboflavin and roseoflavin, a structural analogue of riboflavin, on the growth of selected microbial isolates from human saliva using a riboflavin-free cultivation medium**

Nadja Schwendenmann^1^, Sophie Burger^1^, Leonie Steiner^1^, Lars Seufert^2^, Matthias Kohl^3^, Matthias Mack^2^, Markus Egert*^1^

*^1^Microbiology and Hygiene Group, Institute of Precision Medicine, Furtwangen University of Applied Sciences, Villingen-Schwenningen, Germany*

*^2^Institute of Technical Microbiology, Mannheim University of Applied Sciences, Mannheim, Germany*

*^3^Bioinformatics and Biostatistics Group, Institute of Precision Medicine, Furtwangen University of Applied Sciences, Villingen-Schwenningen, Germany*

*Correspondence*:

* Prof. Dr. Markus Egert, Faculty of Medical and Life Sciences, Institute of Precision Medicine, Microbiology and Hygiene Group, Furtwangen University, Villingen-Schwenningen, Germany. Telephone: +49 7720 3074554, Fax: +49 7720 3074207, E-mail: Markus.Egert@hs-furtwangen.de

## **Supplementary material**

**Online Resource 2** Isolated microorganisms from the human oral cavity, which were added to the proprietary strain collection. Only species that had a MALDI score ≥ 2.0 (secure genus, probable species identification) were included. In total, the proprietary strain collection is comprised of 30 different microorganisms: 1 fungus, 10 gram-negative and 19 gram-positive bacteria. Microorganisms marked with an asterisk (*) could not be identified on species level, but on genus level via MALDI-TOF-MS.

| **Species in alphabetical order** | **Gram stain** | **Habitat** |
| --- | --- | --- |
| *Acinetobacter baumannii* complex *** (Gerner-Smidt, 1992) | negative | Water and soil, skin, oropharynx (Rahal and Urban, 2000), nosocomial pathogen (Howard et al., 2012) |
| *Bacillus subtilis* (DSM 10) | positive | Ubiquitous (Errington and Aart, 2020) |
| *Candida albicans* | - | Gastrointestinal tract (Gow and Yadav, 2017), association with cariogenic *S. mutans* (Sztajer et al., 2014) |
| *Enterococcus faecalis* | positive | Ubiquitous, gastrointestinal tract (Gilmore et al., 2013) |
| *Escherichia coli* | negative | Ubiquitous, gastrointestinal tract (Tenaillon et al., 2010) |
| *Granulicatella adiacens* | positive | Bloodstream (Bouvet et al., 1989; Collins and Lawson, 2000), oral cavity (Sato et al., 1999) |
| *Klebsiella oxytoca* | negative | Ubiquitous, gastrointestinal tract (Bagley, 1985), nosocomial pathogen (Weber-Dąbrowska et al., 2023) |
| *Micrococcus luteus* | positive | Ubiquitous, skin (Lange-Asschenfeldt et al., 2011), upper respiratory tract (Kloos and Musselwhite, 1975) |
| *Moraxella osloensis* | negative | Upper respiratory tract, skin (Berger and Falsen, 1976) |
| *Neisseria mucosa* | negative | Oral cavity (Liu et al., 2015) |
| *Neisseria subflava* group *** (Marri et al., 2010) | negative | Oral cavity (Liu et al., 2015) |
| *Pseudomonas oryzihabitans* | negative | Environmental sources (Kodama et al., 1985) |
| *Rothia mucilaginosa* | positive | Oral cavity (Bergan and Kocur, 1982; Collins et al., 2000) |
| *Schaalia odontolytica* | positive | Oral cavity (Könönen and Wade, 2015) |
| *Staphylococcus aureus* | positive | Ubiquitous, skin, upper respiratory tract (Otto, 2010) |
| *Staphylococcus capitis* | positive | Skin, scalp (Kloos and Schleifer, 1975) |
| *Staphylococcus epidermidis* | positive | Skin, mucosa (Schleifer and Kloos, 1975) |
| *Staphylococcus hominis* | positive | Skin (Kloos and Schleifer, 1975) |
| *Streptococcus anginosus* | positive | Oral cavity, saliva (Kumagai et al., 2003) |
| *Streptococcus cristatus* | positive | Oral cavity (Handley et al., 1991), (Inquimbert et al., 2019) |
| *Streptococcus gordonii* | positive | Skin, oral cavity (Chávez de Paz et al., 2005) |
| *Streptococcus intermedius* | positive | Oral cavity (Whiley and Beighton, 1991) |
| *Streptococcus mitis* group *** | positive | Oral cavity (Hohwy and Kilian, 1995), upper respiratory tract (Human Microbiome Project Consortium, 2012) |
| *Streptococcus mutans* (DSM 20523) | positive | Oral cavity, caries pathogen (Loesche, 1986) |
| *Streptococcus parasanguinis* | positive | Oral cavity (Becker et al., 2002) |
| *Streptococcus salivarius* | positive | Oral cavity (Aas et al., 2005) |
| *Streptococcus sanguinis* | positive | Oral cavity (Kilian et al., 1989) |
| *Veillonella atypica* | negative | Oral cavity (Gibbons and Houte, 1975) |
| *Veillonella dispar* | negative | Oral cavity (Sato et al., 2020) |
| *Veillonella rogosae* | negative | Oral cavity (Arif et al., 2008) |

## **References**

Aas, J.A., Paster, B.J., Stokes, L.N., Olsen, I., Dewhirst, F.E., 2005. Defining the Normal Bacterial Flora of the Oral Cavity. J. Clin. Microbiol. 43, 5721–5732. https://doi.org/10.1128/JCM.43.11.5721-5732.2005

Arif, N., Do, T., Byun, R., Sheehy, E., Clark, D., Gilbert, S.C., Beighton, D., 2008. Veillonella rogosae sp. nov., an anaerobic, Gram-negative coccus isolated from dental plaque. Int. J. Syst. Evol. Microbiol. 58, 581–584. https://doi.org/10.1099/ijs.0.65093-0

Bagley, S.T., 1985. Habitat association of Klebsiella species. Infect. Control IC 6, 52–58. https://doi.org/10.1017/s0195941700062603

Becker, M.R., Paster, B.J., Leys, E.J., Moeschberger, M.L., Kenyon, S.G., Galvin, J.L., Boches, S.K., Dewhirst, F.E., Griffen, A.L., 2002. Molecular analysis of bacterial species associated with childhood caries. J. Clin. Microbiol. 40, 1001–1009. https://doi.org/10.1128/JCM.40.3.1001-1009.2002

Bergan, T., Kocur, M., 1982. NOTES: Stomatococcus mucilaginosus gen.nov., sp.nov., ep. rev., a Member of the Family Micrococcaceae. Int. J. Syst. Evol. Microbiol. 32, 374–377. https://doi.org/10.1099/00207713-32-3-374

Berger, U., Falsen, E., 1976. über die Artenverteilung vonMoraxella und moraxella-ähnlichen Keimen im Nasopharynx gesunder Erwachsener. Med. Microbiol. Immunol. (Berl.) 162, 239–249. https://doi.org/10.1007/BF02121002

Bouvet, A., Grimont, F., Grimont, P.A.D., 1989. Streptococcus defectivus sp. nov. and Streptococcus adjacens sp. nov., Nutritionally Variant Streptococci from Human Clinical Specimens. Int. J. Syst. Evol. Microbiol. 39, 290–294. https://doi.org/10.1099/00207713-39-3-290

Chávez de Paz, L., Svensäter, G., Dahlén, G., Bergenholtz, G., 2005. Streptococci from root canals in teeth with apical periodontitis receiving endodontic treatment. Oral Surg. Oral Med. Oral Pathol. Oral Radiol. Endod. 100, 232–241. https://doi.org/10.1016/j.tripleo.2004.10.008

Collins, M.D., Hutson, R.A., Båverud, V., Falsen, E., 2000. Characterization of a Rothia-like organism from a mouse: description of Rothia nasimurium sp. nov. and reclassification of Stomatococcus mucilaginosus as Rothia mucilaginosa comb. nov. Int. J. Syst. Evol. Microbiol. 50, 1247–1251. https://doi.org/10.1099/00207713-50-3-1247

Collins, M.D., Lawson, P.A., 2000. The genus Abiotrophia (Kawamura et al.) is not monophyletic: proposal of Granulicatella gen. nov., Granulicatella adiacens comb. nov., Granulicatella elegans comb. nov. and Granulicatella balaenopterae comb. nov. Int. J. Syst. Evol. Microbiol. 50, 365–369. https://doi.org/10.1099/00207713-50-1-365

Errington, J., Aart, L.T. van der, 2020. Microbe Profile: Bacillus subtilis: model organism for cellular development, and industrial workhorse. Microbiology 166, 425–427. https://doi.org/10.1099/mic.0.000922

Gerner-Smidt, P., 1992. Ribotyping of the Acinetobacter calcoaceticus-Acinetobacter baumannii complex. J. Clin. Microbiol. 30, 2680–2685. https://doi.org/10.1128/jcm.30.10.2680-2685.1992

Gibbons, R.J., Houte, J.V., 1975. Bacterial adherence in oral microbial ecology. Annu. Rev. Microbiol. 29, 19–42. https://doi.org/10.1146/annurev.mi.29.100175.000315

Gilmore, M.S., Lebreton, F., van Schaik, W., 2013. Genomic transition of enterococci from gut commensals to leading causes of multidrug-resistant hospital infection in the antibiotic era. Curr. Opin. Microbiol. 16, 10–16. https://doi.org/10.1016/j.mib.2013.01.006

Gow, N.A.R., Yadav, B., 2017. Microbe Profile: Candida albicans: a shape-changing, opportunistic pathogenic fungus of humans: This article is part of the Microbe Profiles collection. Microbiology 163, 1145–1147. https://doi.org/10.1099/mic.0.000499

Handley, P., Coykendall, A., Beighton, D., Hardie, J.M., Whiley, R.A., 1991. Streptococcus crista sp. nov., a viridans streptococcus with tufted fibrils, isolated from the human oral cavity and throat. Int. J. Syst. Bacteriol. 41, 543–547. https://doi.org/10.1099/00207713-41-4-543

Hohwy, J., Kilian, M., 1995. Clonal diversity of the Streptococcus mitis biovar 1 population in the human oral cavity and pharynx. Oral Microbiol. Immunol. 10, 19–25. https://doi.org/10.1111/j.1399-302X.1995.tb00113.x

Howard, A., O’Donoghue, M., Feeney, A., Sleator, R.D., 2012. Acinetobacter baumannii. Virulence 3, 243–250. https://doi.org/10.4161/viru.19700

Human Microbiome Project Consortium, 2012. Structure, Function and Diversity of the Healthy Human Microbiome. Nature 486, 207–214. https://doi.org/10.1038/nature11234

Inquimbert, C., Bourgeois, D., Bravo, M., Viennot, S., Tramini, P., Llodra, J.C., Molinari, N., Dussart, C., Giraudeau, N., Carrouel, F., 2019. The Oral Bacterial Microbiome of Interdental Surfaces in Adolescents According to Carious Risk. Microorganisms 7, 319. https://doi.org/10.3390/microorganisms7090319

Kilian, M., Kißling, L., Henrichsen, J., 1989. Taxonomic Study of Viridans Streptococci: Description of Streptococcus gordonii sp. nov. and Emended Descriptions of Streptococcus sanguis (White and Niven 1946), Streptococcus oralis (Bridge and Sneath 1982), and Streptococcus mitis (Andrewes and Horder 1906). Int. J. Syst. Evol. Microbiol. 39, 471–484. https://doi.org/10.1099/00207713-39-4-471

Kloos, W.E., Musselwhite, M.S., 1975. Distribution and Persistence of Staphylococcus and Micrococcus Species and Other Aerobic Bacteria on Human Skin1. Appl. Microbiol. 30, 381–395.

Kloos, W.E., Schleifer, K.H., 1975. Isolation and Characterization of Staphylococci from Human Skin II. Descriptions of Four New Species: Staphylococcus warneri, Staphylococcus capitis, Staphylococcus hominis, and Staphylococcus simulans1. Int. J. Syst. Evol. Microbiol. 25, 62–79. https://doi.org/10.1099/00207713-25-1-62

Kodama, K., Kimura, N., Komagata, K., 1985. Two New Species of Pseudomonas: P. oryzihabitans Isolated from Rice Paddy and Clinical Specimens and P. luteola Isolated from Clinical Specimens. Int. J. Syst. Evol. Microbiol. 35, 467–474. https://doi.org/10.1099/00207713-35-4-467

Könönen, E., Wade, W.G., 2015. Actinomyces and Related Organisms in Human Infections. Clin. Microbiol. Rev. 28, 419–442. https://doi.org/10.1128/CMR.00100-14

Kumagai, K., Sugano, N., Takane, M., Iwasaki, H., Tanaka, H., Yoshinuma, N., Suzuki, K., Ito, K., 2003. Detection of Streptococcus anginosus from saliva by real-time polymerase chain reaction. Lett. Appl. Microbiol. 37, 370–373. https://doi.org/10.1046/j.1472-765X.2003.01405.x

Lange-Asschenfeldt, B., Marenbach, D., Lang, C., Patzelt, A., Ulrich, M., Maltusch, A., Terhorst, D., Stockfleth, E., Sterry, W., Lademann, J., 2011. Distribution of bacteria in the epidermal layers and hair follicles of the human skin. Skin Pharmacol. Physiol. 24, 305–311. https://doi.org/10.1159/000328728

Liu, G., Tang, C.M., Exley, R.M., 2015. Non-pathogenic Neisseria: members of an abundant, multi-habitat, diverse genus. Microbiol. Read. Engl. 161, 1297–1312. https://doi.org/10.1099/mic.0.000086

Loesche, W.J., 1986. Role of Streptococcus mutans in human dental decay. Microbiol. Rev. 50, 353–380.

Marri, P.R., Paniscus, M., Weyand, N.J., Rendón, M.A., Calton, C.M., Hernández, D.R., Higashi, D.L., Sodergren, E., Weinstock, G.M., Rounsley, S.D., So, M., 2010. Genome Sequencing Reveals Widespread Virulence Gene Exchange among Human Neisseria Species. PLoS ONE 5, e11835. https://doi.org/10.1371/journal.pone.0011835

Otto, M., 2010. Staphylococcus colonization of the skin and antimicrobial peptides. Expert Rev. Dermatol. 5, 183–195. https://doi.org/10.1586/edm.10.6

Rahal, J.J., Urban, C., 2000. Acinetobacter. Semin. Respir. Crit. Care Med. 21, 341–348. https://doi.org/10.1055/s-2000-9858

Sato, N., Kakuta, M., Hasegawa, T., Yamaguchi, R., Uchino, E., Kobayashi, W., Sawada, K., Tamura, Y., Tokuda, I., Murashita, K., Nakaji, S., Imoto, S., Yanagita, M., Okuno, Y., 2020. Metagenomic analysis of bacterial species in tongue microbiome of current and never smokers. NPJ Biofilms Microbiomes 6, 11. https://doi.org/10.1038/s41522-020-0121-6

Sato, S., Kanamoto, T., Inoue, M., 1999. Abiotrophia elegans strains comprise 8% of the nutritionally variant streptococci isolated from the human mouth. J. Clin. Microbiol. 37, 2553–2556. https://doi.org/10.1128/JCM.37.8.2553-2556.1999

Schleifer, K.H., Kloos, W.E., 1975. Isolation and Characterization of Staphylococci from Human Skin I. Amended Descriptions of Staphylococcus epidermidis and Staphylococcus saprophyticus and Descriptions of Three New Species: Staphylococcus cohnii, Staphylococcus haemolyticus, and Staphylococcus xylosus. Int. J. Syst. Evol. Microbiol. 25, 50–61. https://doi.org/10.1099/00207713-25-1-50

Sztajer, H., Szafranski, S.P., Tomasch, J., Reck, M., Nimtz, M., Rohde, M., Wagner-Döbler, I., 2014. Cross-feeding and interkingdom communication in dual-species biofilms of Streptococcus mutans and Candida albicans. ISME J. 8, 2256–2271. https://doi.org/10.1038/ismej.2014.73

Tenaillon, O., Skurnik, D., Picard, B., Denamur, E., 2010. The population genetics of commensal Escherichia coli. Nat. Rev. Microbiol. 8, 207–217. https://doi.org/10.1038/nrmicro2298

Weber-Dąbrowska, B., Żaczek, M., Łobocka, M., Łusiak-Szelachowska, M., Owczarek, B., Orwat, F., Łodej, N., Skaradzińska, A., Łaczmański, Ł., Martynowski, D., Kaszowska, M., Górski, A., 2023. Characteristics of Environmental Klebsiella pneumoniae and Klebsiella oxytoca Bacteriophages and Their Therapeutic Applications. Pharmaceutics 15, 434. https://doi.org/10.3390/pharmaceutics15020434

Whiley, R.A., Beighton, D., 1991. Emended Descriptions and Recognition of Streptococcus constellatus, Streptococcus intermedius, and Streptococcus anginosus as Distinct Species. Int. J. Syst. Bacteriol. 41, 1–5. https://doi.org/10.1099/00207713-41-1-1
